# WNT4 secreted by tumor tissues promotes tumor progression in colorectal cancer by activation of the Wnt/β-catenin signalling pathway

**DOI:** 10.1186/s13046-020-01774-w

**Published:** 2020-11-23

**Authors:** Dongmei Yang, Qing Li, Renduo Shang, Liwen Yao, Lianlian Wu, Mengjiao Zhang, Lihui Zhang, Ming Xu, Zihua Lu, Jie Zhou, Li Huang, Xiaodong Huang, Du Cheng, Yanning Yang, Honggang Yu

**Affiliations:** 1 Department of Gastroenterology, Renmin Hospital of Wuhan University, Wuhan, 430060 China; 2 Hubei Key Laboratory of Digestive System Disease, Renmin Hospital of Wuhan University, Wuhan, 430060 China; 3 Hubei Provincial Clinical Research Center for Digestive Disease Minimally Invasive Incision, Renmin Hospital of Wuhan University, Jiefang Rd. 238, Wuhan, 430060 China; 4 Department of Gastrointestinal Surgery, Renmin Hospital of Wuhan University, Wuhan, 430060 China; 5 Department of Ophthalmology, Renmin Hospital of Wuhan University, Jiefang Rd. 238, Wuhan, Hubei 430060 People’s Republic of China

**Keywords:** WNT4, Biomarker, Colorectal cancer, Tumor metastasis, Epithelial-mesenchymal transition, Fibroblast, Angiogenesis

## Abstract

**Background:**

Wingless and Int-related protein (Wnt) ligands are aberrantly expressed in patients with colorectal cancer (CRC). However, the aberrant level of Wnt ligands in serum have not been explored. Here, we aimed to identify the levels of WNT4 in serum and explored its oncogenic role in CRC.

**Methods:**

The Oncomine database was used to analyze the relationship between WNT4 and the prognosis of CRC. ELISA was performed to measure WNT4 levels in serum and conditioned medium from fresh CRC tissues and adjacent normal tissues. Western blot and immunohistochemistry were carried out to measure the expression of WNT4 in human CRC tissues and adjacent normal tissues. The migration and invasion of CRC cells were determined by trans-well assay, and the effects of WNT4 on CRC invasion and metastasis in vivo were verified by tumor xenograft in nude mice. Cancer-associated fibroblasts (CAFs) and angiogenesis in subcutaneous nodules were detected by immunofluorescence (IF). In addition, the suspended spheres formation and tube formation assay were performed to explore the effects of WNT4 on CAFs and angiogenesis respectively.

**Results:**

WNT4 was significantly upregulated in serum of CRC patients, and CRC tissues were identified as an important source of elevated WNT4 levels in CRC patients. Interestingly, elevated levels of WNT4 in serum were downregulated after tumor resection. Furthermore, we found that WNT4 contributed to epithelial-to-mesenchymal transition (EMT) and activated fibroblasts by activating the WNT4/β-catenin pathway in vitro and in vivo. Moreover, angiogenesis was induced via the WNT4/β-catenin/Ang2 pathway. Those effects could be reversed by ICG-001, a β-catenin/TCF inhibitor.

**Conclusion:**

Our findings indicated that serum levels of WNT4 may be a potential biomarker for CRC. WNT4 secreted by colorectal cancer tissues promote the progression of CRC by inducing EMT, activate fibroblasts and promote angiogenesis through the canonical Wnt/β-catenin signalling pathway.

**Supplementary Information:**

The online version contains supplementary material available at 10.1186/s13046-020-01774-w.

## Background

Colorectal cancer (CRC) is one of the leading causes of cancer-related deaths worldwide [1], and evolves as a result of the accumulation of genetic and epigenetic events [2]. Data evaluated by the International Agency for Research on Cancer (IARC) indicated that about 1.8 million new cases of CRC were diagnosed and over 860,000 CRC patients died worldwide in 2018, which account for about 10% of all cancers and 9% of all cancer-related deaths, respectively [3]. Therefore, it is of most importance to develop sensitive biomarkers and effective treatments for patients with CRC.

Nowadays, increased attention has been paid to the research of biomarkers for diseases, which could be used for early diagnosis and predict cancer outcome [4]. Moreover, measuring biomarkers in blood offers a simple and effective way for disease diagnosis [5]. Among nineteen identified Wingless and Int-related protein (WNT) ligands, WNT4 is highly secretory and its secretion has been reported in wound healing, acute kidney injury, and angiogenesis [6]. However, to our knowledge, no study has resported on exploring Wnt levels in blood. In our study, we aimed to study WNT4 levels in serum, and explored its oncogenic role in CRC.

The tumor stroma is composed of extra-cellular matrix (ECM) proteins and a variety of cell types including fibroblasts, infiltrating immune cells, and endothelial cells [7, 8]. Fibroblasts are a major component of tumor stroma and may acquire an activated phenotype called cancer associated fibroblasts (CAFs), which specifically express markers, such as fibronectin (FN) and α-SMA [9, 10]. CAF has been demonstrated to have the ability to modulate the tumor microenvironment to promote CRC [11]. Cytokines and inflammatory factors derived from cancer tissue can stimulate the transformation of normal fibroblasts (NFs) into CAFs [12]. Therefore, identifying activators of CAFs in CRC may potentially help inhibit the development of CRC.

In addition, tumor start to grow only when the endothelial cells are recruited to the tumor site and form capillaries [13]. Previous studies have demonstrated that angiogenesis could be enhanced by the WNT4/β-catenin pathway in Human umbilical cord mesenchymal stem cells [14]. ANG2 is a crucial gene that is vital for angiogenesis and is closely related to the functions of human umbilical vein endothelial cells (HUVECs) [15]. However, it is not clear if WNT4 can affect angiogenesis in CRC by regulating ANG2.

The Wnt/β-catenin pathway is the “canonical” Wnt pathway, which is essential for stem cell self-renewal and maintaining homeostasis of the intestinal tract [16]. As a member of Wnt family, WNT4 may play a role in CRC through the β-catenin-dependent pathway. Therefore, it would be interesting to elucidate whether WNT4 can promote the progression of CRC via promoting epithelial-to-mesenchymal transition (EMT) of CRC cells, activating CAFs and enhancing angiogenesis through the β-catenin-dependent pathway.

In this study, we first showed that WNT4 levels were increased in the serum of CRC patients and originated from CRC tissues, and were decreased after tumor resection. Mechanistically, we explored the effect of WNT4 on the development of CRC in vitro and in vivo*,* we found that WNT4 promoted EMT of CRC cells, triggered the transformation of NFs into CAFs, and induced tumors angiogenesis in a β-catenin-dependent pathway.

## Materials and methods

### Human colorectal tissue and serum samples

The CRC tissues and paired adjacent normal tissues were collected from 24 CRC patients undergoing surgical resection (8 were used for Western blot analysis, 2 were used for the isolation of NFs and CAFs, 4 were used for tissue culture and another 10 were used for qPCR analysis). Serum samples from 40 CRC patients and 37 healthy donors were also collected at the Renmin Hospital of Wuhan University, Wuhan, China. Informed consents were signed by those patients and healthy donors before sample collection. All works were approved by the ethics review board of Renmin Hospital of Wuhan University (Wuhan, China) and carried out in compliance with the Declaration of Helsinki of the World Medical Association.

### Oncomine database analysis

The Oncomine gene expression array database (www.oncomine.org) was used to assess mRNA levels of WNT4 in two CRC datasets, including Sabates-Bellver Colon (http://www.ncbi.nlm.nih.gov/geo/query/acc.cgi?acc=GSE8671) and Skrzypczak Colorectal (http://www.ncbi.nlm.nih.gov/geo/query/acc.cgi?acc=GSE20916). The Cancer Genome Atlas (TCGA; http://tcga-data.nci.nih.gov/tcga/) Colorectal and Jorissen Colorectal 3 (http://www.ncbi.nlm.nih.gov/geo/query/acc.cgi?acc=GSE14333) were applied to analyze survival rates and recurrence rates respectively. Samples with incomplete clinical information were excluded.

### Antibodies and reagents

Antibodies were obtained from the following sources: rabbit anti-WNT4 polyclonal antibody (ab91226), rabbit anti-AXIN2 monoclonal antibody (ab109307), rabbit anti-fibronectin polyclonal antibody (ab2413) and rabbit anti-β-catenin monoclonal antibody (ab32572) were obtained from Abcam (Cambridge, MA, USA); rabbit anti-ZO-1 monoclonal antibody (#13663) and rabbit anti-E-cadherin monoclonal antibody (#3195) were obtained from Cell Signaling Technology (Beverly, MA, USA); anti-rabbit and anti-mouse HRP-conjugated secondary antibodies (#31460, #31430) were obtained from Invitrogen (Carlsbad, CA, USA). Anti-fluorescein isothiocyanate labeled phalloidin (P5282) and anti-α-SMA mouse monoclonal (A5228) antibodies were obtained from Sigma-Aldrich (St. Louis, MO, USA). Recombinant Human WNT4 Protein (6076-WN-005/CF) was obtained from R&D Systems (Minneapolis, MN, USA).

### Cell culture

The CRC cell lines HCT 116, LoVo and SW480 were cultured in Dulbecco’s modified Eagle medium (DMEM, Gibco, Carlsbad, CA, USA) supplemented with 10% fetal bovine serum (FBS), 100 U/mL penicillin, and 100 mg/mL streptomycin. HUVECs were cultured in Ham’s F-12 K (HyClone, Thermo Fisher Scientific, Waltham, MA, USA) supplemented with 10% FBS, 0.1 mg/mL heparin, 0.03–0.05 mg/ml endothelial cell growth supplement (ECGs). Cells were incubated in a humidified incubator containing 5% CO_2_ at 37 °C.

### Immunohistochemical staining

Indirect immunohistochemical staining was performed as follows. All sections were dewaxed and dehydrated, followed by antigen retrieval and blocking. Subsequently, sections were incubated with a primary antibody and HRP-conjugated secondary antibodies. A 3,3′-diaminobenzidine (DAB) kit was used for visualization, and hematoxylin was used to stain the nuclei. Finally, sections were dehydrated with alcohol and sealed with neutral resin.

### Cell migration and invasion assay

The cell migration assay was performed using chambers (8.0 μm) in a 24-well plate. For migration assays, cells pretreated with 100 ng/mL WNT4 for 24 h or not were added to the upper chamber, which contained serum-free DMEM; the lower compartment was filled with DMEM medium supplemented with 10% FBS. Cells were allowed to migrate for 12 h, and those that had migrated across the filter were fixed in methanol and stained with 0.1% crystal violet. The invasion potential of those cells were determined by using a trans-well chamber (8.0 μm), coated with Matrigel (Corning, Albany, NY, USA) in DMEM medium (1:4 v/v), and the lower compartment was filled with DMEM medium supplemented with 20% FBS. Cells were incubated for 24 h before invasive cells were fixed, stained, and counted. Results were analyzed by ImageJ.

### ELISA

The WNT4 concentration in serum from healthy donors, CRC patients, and conditioned medium from fresh tissues were quantitatively determined by ELISA kits (CSB-EL026137HU, CUSABIO, Wuhan, China) according to the manufacturer’s instructions. For ANG2, the protein concentrations in media from HUVEC cells were detected by a human ANG2 ELISA kit (Abcam, Cambridge, MA, USA).

### Luciferase assay

A Dual-Luciferase Reporter Assay system was used to measure luciferase activities (Promega, Fitchburg, WI, USA). For TOP/FOP-Flash reporter assays, cells were divided into five groups: blank, WNT4 (100 ng/mL), DMSO, ICG-001, and ICG-001+ WNT4 (100 ng/mL). After transfection, WNT4 protein (100 ng/mL) was added 6 h before luciferase detection. The relative ratio of TOP-Flash firefly luciferase activity to pRL-TK *Renilla* luciferase activity was determined as the strength of the transcriptional activity.

### Western blots

In brief, equal amounts of protein were separated using SDS–PAGE, and transferred onto PVDF membranes (Millipore, Burlington, MA, USA). After blocking with 5% non-fat milk in Tris-buffered saline with Tween 20 (TBST), membranes were incubated with primary antibodies at 4 °C overnight and HRP-conjugated secondary antibodies at room temperature for 60 min. Then membrane were incubated with enhanced chemiluminescence (ECL) reagents, and signals were visualized by chemiluminescent a gel imaging system.

### Immunofluorescence

For immunofluorescence assays, 12-well chambers were used. Each well was seeded with 1 × 10^5^ cells on the coverslip and fixed with 4% paraformaldehyde, treated with 0.2% Triton X-100 for 1 min and blocked with 1% bovine serum albumin (BSA). For paraffin embedded tissues, sections were dewaxed and dehydrated, followed by antigen retrieval and blocking. After incubation with primary antibodies and fluorescent-labeled secondary antibodies and Phalloidin, the cells on coverslips or tissue sections were stained with 4′,6-diamidino-2-phenylindole (DAPI). Fluorescent images were obtained using an upright Olympus fluorescence microscope (OLYMPUS BX53).

### Cell transfection

Inhibition of WNT4 expression was performed by using specific siRNA constructs. The sequences of WNT4 siRNA-1, WNT4 siRNA-2 and normal control used in this study are presented in Supplementary Table 1. In brief, cells were transiently transfected with WNT4 siRNAs using Lipofectamine 2000 and were harvested for analysis of the knockdown efficiency at 48 h ours after transfection.

To establish stable WNT4-overexpressing cells, an overexpressing plasmid was constructed and packaged into a lentivirus system (WNT4-HA). Lentivirus-NC was used as the negative control (WNT4-vector). In addition, to generate cells with low expression of WNT4 (sh-WNT4) and ANG2 (sh-ANG2), short hairpin RNA (shRNA) sequences directed against WNT4 and ANG2 were transfected (Supplementary Table 1), while a scramble construct was used as a control. Non-transfected cells were screened out by treatment with 10 μg/mL puromycin (Sigma Aldrich, St Louis, MO).

### Quantitative real-time PCR

TRIzol reagents (Invitrogen) was used to extract RNA from cell lines or tissues according to the manufacturer’s instructions. RT-qPCR was performed to quantify the mRNA expression using SYBR Green PCR Master Mix (Takara, Ohtsu, Japan), and miRNA expression with the NCode miRNA RT-qPCR analysis (Takara, Ohtsu, Japan). Primer sequences are presented in Supplementary Table 2.

### Tumor xenograft study

To generate a xenograft model, 1 × 10^7^ cells/200 μL were injected subcutaneously into the dorsal flank of each mouse (8-week-old, male, BALB/c-nu/nu). After 2 weeks, mice were sacrificed, and subcutaneous nodules were collected. For establishing the tumor metastasis model, 1 × 10^6^ cells/100 μL were injected into the tail vein of each mouse (8-week-old, male, BALB/c-nu/nu). After 4 weeks, mice were sacrificed, and the weights of mice livers were recorded.

After tumor weights were recorded, all tissues were fixed with 4% paraformaldehyde. Animal experiments were approved by the Committee on the Use of Live Animals in Teaching and Research (CULATR), Renmin Hospital of Wuhan University (No:11400700326686 and No:110011111003022). Animals care was in accordance with institution guidelines.

### Isolation of NFs and CAFs from CRC and normal tissues

Fresh tissues were cut into small pieces (around 1 mm^3^) and subjected to enzymatic digestion in DMEM supplemented with 5% FBS following the instructions of the tumor dissociation kit (Miltenyi Biotec, Bergisch Gladbach, Germany). Next, cells were filtered through a 100-μm cell strainer (Thermo Fisher Scientific, MA, USA) and resuspended and cultured in fibroblast medium (FM). Flow cytometry using a CD31-Cy7 conjugated antibody, CD45-Cy7 conjugated antibody, and CD329-Cy7 conjugated antibody was performed to confirm the absence of endothelial, immune, and epithelial cell contamination in the primary fibroblasts.

### Preparation of conditioned medium

Fresh human CRC and adjacent normal tissue samples were obtained directly from the operating room. Tissues were weighed and preserved in Falcon tubes containing 10 mL of DMEM with antibiotics (100 U/mL penicillin, and 100 mg/mL streptomycin) to avoid bacterial or fungal contamination. Tissues were cut into similar sized pieces and cultured with DMEM for 12 h. Then, collected conditioned medium (CM) was collected, and filtered through 40-μm cell strainers (Thermo Fisher Scientific, MA, USA) to obtain tissue-conditioned medium and stored at − 80 °C until further use.

After the SW480 cells (vector/WNT4-HA) grew to 70–80% confluency, then cells cultured in DMEM medium (no FBS) for 24 h. CM was obtained and centrifuged (1000 rpm, 10 min), and the supernatant was filtered through a 0.22 μm filter (Beyotime Biotechnology, China) and stored at 4 °C for treating HUVECs.

### Tube formation assay

The tube formation assay was performed following the manufacturer’s protocol (BD Biosciences, Franklin Lakes, NJ, USA, https://www.bdbiosciences.com). In brief, 50 μL of growth factor reduced Matrigel (BD Biosciences company, San Jose, CA, USA) was added to each well of a precooled 96-well plate and allowed to polymerize at 37 °C. Subsequently, HUVECs or transfected HUVECs (sh-ANG2/scramble ANG2) were treated with CM from SW480 cells (vector/WNT4-HA) and pretreated with ICG-001 (10 μM) 12 h before collection. Subsequently, cell (50 μL) at a density of 1 × 10^5^ cells/mL were seeded onto the matrix gel and incubated at 37 °C for 12 h. Then, cells were viewed under a microscope (OLYMPUS IX71, Japan) and imaged. The tube length was measured by ImageJ software (https://imagej.nih.gov/ij/, MD, USA).

### Suspended spheres formation

The protocol used for analyzing sphere formation was described previously [17]. In brief, primary fibroblasts were labeled with PKH-26 (red) and mixed with GFP-transfected tumor cells (Scramble or sh-WNT4, WNT4-vector or WNT4-HA) at a ratio of 3:1 in an ultra-low attachment plate (Corning, Albany, NY, USA) with DMEM medium at 37 °C overnight. Typical heterospheroids were observed and counted using an inverted Olympus fluorescence microscope (OLYMPUS IX71).

### Collagen matrix contraction assay

Contraction of collagen gels was performed in 96-well plates as previously reported [18]. In brief, collagen gel (Corning, Albany, NY, USA), matrigel (Corning, Albany, NY, USA), FM, FBS, and suspended fibroblasts (with or without 400 ng/mL WNT4 pretreatment) were gently mixed. Then, 100 μl of the mixture was added to each well of a 96-well plate and allowed to solidify at 37 °C for 30 min before complete medium was added. For inhibitory experiments, both gels and media were incubated with 10 μM ICG-001.

### Statistical analysis

All experiments were performed at least in triplicate. Data are presented as the mean ± standard deviation (SD). Data of the relationship of WNT4, CA199, and CEA expression with clinicopathological parameters of CRC patients are presented as the mean ± standard error of mean (SEM). Comparisons between two groups were performed by Student’s t-tests or Mann–Whitney U tests for continuous variables. Survival probabilities and recurrence rates were estimated using Kaplan–Meier survival analysis and differences between Kaplan–Meier curves were compared by the log-rank test. All statistical tests were two-sided. SPSS v17.0 (Chicago, IL, USA) was used for statistical analyses. *P* < 0.05 were considered statistically significant. *P < 0.05, **P < 0.01.

## Results

### WNT4 was upregulated in serum and cancer tissues of CRC patients

**Fig. 1:**
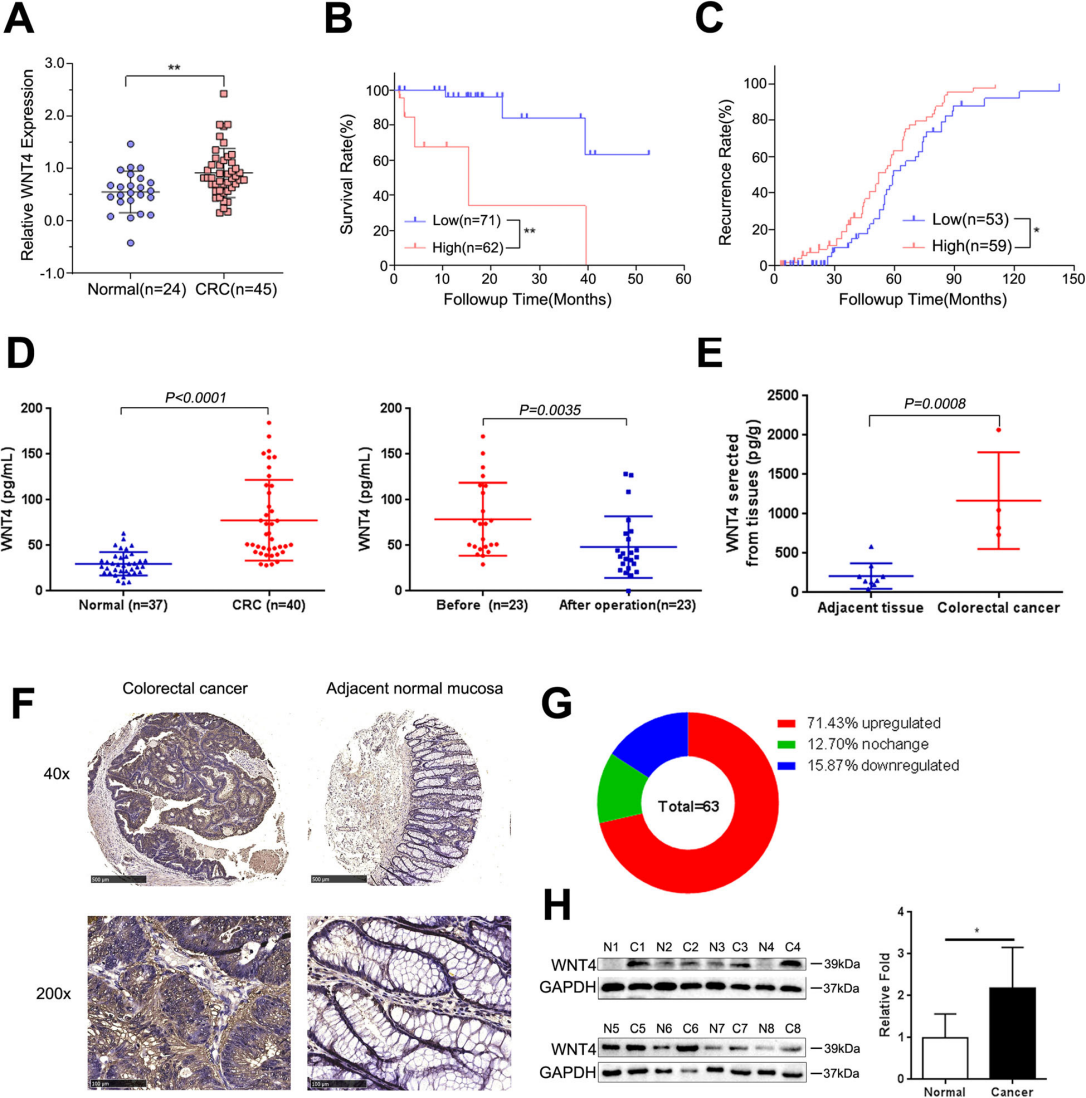
WNT4 was upregulated in CRC. **a** WNT4 expression level in colorectal cancer (CRC) tissue (*n* = 45) and normal tissue (*n* = 24) from the Oncomine Database. **b** and **c** CRC patients with higher WNT4 expression had lower survival rate and higher recurrence rate from The Cancer Genome Atlas Colorectal (low WNT4 group, *n* = 71; high WNT4 group, *n* = 62) and Jorissen Colorectal 3 (low WNT4 group, *n* = 53; high WNT4 group, *n* = 59) respectively. High expression of WNT4 is defined as greater than the median value, and low expression is defined as less than or equal to the median value. **d** The serum level of WNT4 in CRC patients were higher than that in healthy donors, while it was decreased after radical surgery in 21/23 (91.30%) of CRC patients. **e** WNT4 levels secreted from per gram of CRC tissues and adjacent tissue after overnight culture. **f**, **g** and **h** The expression of WNT4 was significantly higher in CRC tissue compared to the adjacent tissue in immunohistochemistry and western blots. Upregulation was defined as WNT4 expression was higher in CRC tissues compared to that in paired normal tissues, while downregulation was defined as WNT4 expression was lower in CRC tissues compared to that in paired normal tissues. Data are presented as the mean ± SD. Two-tailed Student’s t test was used for statistical analyses. *P < 0.05, **P < 0.01

We first analyzed the expression of WNT4 in CRC tissues and normal tissues from the Oncomine database. Our data showed that WNT4 was elevated in CRC patients (Fig. 1a, *P* < 0.05). Moreover, CRC patients with a high WNT4 expression had a lower survival rate and a higher recurrence rate compared to CRC patients with a low WNT4 expression (Fig. 1b, c, *P* < 0.05). In addition, tissues and serum samples were collected from CRC patients and healthy donors. ELISA was employed to measure the serum level of WNT4 from 40 CRC patients and 37 healthy donors, and significantly higher WNT4 levels were found in CRC patients compared to healthy donors (Fig. 1d, *P* < 0.01). Interestingly, WNT4 levels were decreased after radical surgery in 21/23 (91.30%) of CRC patients who underwent surgery treatment to remove the tumor (Fig. 1d, *P* < 0.05). Subsequently, CM from fresh tumor and adjacent normal tissues was collected to identify the source of WNT4 hypersecretion. The results showed that WNT4 levels in CM from fresh tumors were significantly higher compared to those from adjacent tissues, which strongly indicated that WNT4 may be derived from CRC tissue (Fig. 1e, *P* < 0.05). Thus, these results may indicate that the increased serum levels of WNT4 in serum were secreted by CRC tumor tissues, which may also explain the decrease of WNT4 in serum after tumor resection. Furthermore, these results suggested that the serum level of WNT4 may be a potential biomarker for CRC.

We next measured the expression of WNT4 in human CRC tissues and paired adjacent normal tissues by immunohistochemistry. Compared with adjacent normal tissues, WNT4 was significantly increased in CRC tissues (Fig. 1f). Immunohistochemical analysis of WNT4 in tumor tissue arrays indicated that the level of WNT4 was elevated in 45/63 (71.43%) of tumors (Fig. 1g). The expression of WNT4 in 8 pairs of freshly frozen CRC tissues and adjacent normal tissues were evaluated by using western blot analysis, and showed that WNT4 was upregulated in CRC tissue (Fig. 1h, *P* < 0.05).

Taken together, these results suggested that WNT4 was upregulated in serum and tissue from CRC patients, and may play a role in the progression of CRC. Combined with the above findings that WNT4 levels decreased after tumor resection, we speculated that the increased levels of WNT4 in serum may be secreted by CRC tumor tissues.

### WNT4 level correlated with metastasis of CRC

Next, we analyzed the relationships between elevated serum levels of WNT4 and clinicopathological characteristics of CRC patients. As shown in Table 1, significantly different WNT4 expression was observed when comparing advanced TNM T stage with early stage (88.08 pg/mL vs. 55.94 pg/mL; *P* = 0.0293), and advanced TNM stage with early stage (94.57 pg/mL vs. 66.35 pg/mL; *P* = 0.0464). In addition, higher levels of WNT4 were found in large tumor size (96.60 pg/mL vs. 59.38 pg/mL; *P* = 0.0087) and tumor with metastasis (111.1 pg/mL vs. 71.73 pg/mL; *P* = 0.0428), while no relationship was observed between WNT4 expression and age, sex, lymph node metastasis, and differentiation (*P* > 0.05). Remarkably, in our samples, according to TNM staging of CRC patients, higher expression of WNT4 was observed with advanced stages (III and IV) compared with earlier stages (I and II) (94.57 ± 11.61 vs. 66.35 ± 8.097; *P* = 0.0464), while CA199 and CEA were not (Table 1). These results indicated that high expression of WNT4 is associated with faster CRC enlarging and spreading, thereby suggesting that the WNT4 level correlated with CRC metastasis.

**Table 1:** Relation of WNT4, CA199, CEA expression with clinicopathological parameters of patients with CRC

Parameters	WNT4 (pg/mL)		*P* value	CA199 (U/mL)		*P* value	CEA (ng/mL)		*P* value
	Cases	Mean ± SEM		Cases	Mean ± SEM		Cases	Mean ± SEM	
Age(y)									
≤ 65	23	77.31 ± 10.04	0.9583	22	31.51 ± 10.27	0.3766	22	4.304 ± 1.315	0.7307
> 65	17	78.07 ± 9.623		16	19.78 ± 5.963		16	3.671 ± 1.143	
Sex									
Male	20	88.46 ± 11.14	0.1129	19	28.36 ± 9.941	0.7862	19	4.445 ± 1.394	0.6533
Female	20	66.81 ± 7.998		19	24.79 ± 8.484		19	3.629 ± 1.141	
TNM T									
I ∼ II	13	55.94 ± 8.395	0.0293	13	26.84 ± 12.39	0.9768	12	1.135 ± 0.237	0.0247
III ∼ IV	27	88.08 ± 8.941		25	26.44 ± 7.594		26	5.377 ± 1.216	
TNM N									
0	26	73.99 ± 9.211	0.4849	26	28.20 ± 8.763	0.7158	25	2.732 ± 0.769	0.0403
1–2	14	84.39 ± 10.49		12	23.05 ± 7.982		13	6.548 ± 2.021	
TNM M									
0	34	71.73 ± 6.868	0.0428	32	21.28 ± 5.175	0.0570	32	3.562 ± 0.76	0.2221
1	6	111.1 ± 22.62		6	54.80 ± 29.61		6	6.575 ± 4.071	
TNM stage									
Early(I/ II)	24	66.35 ± 8.097	0.0464	24	22.71 ± 6.797	0.4407	23	2.710 ± 0.836	0.0642
Advanced (III/IV)	16	94.57 ± 11.61		14	33.20 ± 13.27		15	6.072 ± 1.772	
Grade									
WD	8	62.62 ± 14.33	0.5367	8	37.70 ± 19.55	0.6844	8	1.52 ± 0.41	0.3173
MD	28	82.48 ± 8.83		27	23.70 ± 7.14		26	4.89 ± 1.24	
PD	4	73.76 ± 16.26		3	22.84 ± 3.32		4	3.50 ± 1.59	
Tumor size									
⩽3 cm	18	59.38 ± 8.190	0.0087	17	25.53 ± 9.579	0.8158	17	3.853 ± 1.725	0.7530
> 3 cm	20	96.60 ± 10.38		19	28.77 ± 9.850		19		

Statistical significance was defined as *P* < 0.05

*WD* Well differentiated, *MD* Moderately differentiated, *PD* Poorly differentiated

### WNT4 promotes invasion and migration of CRC through the β-catenin-dependent pathway in vitro

**Fig. 2:**
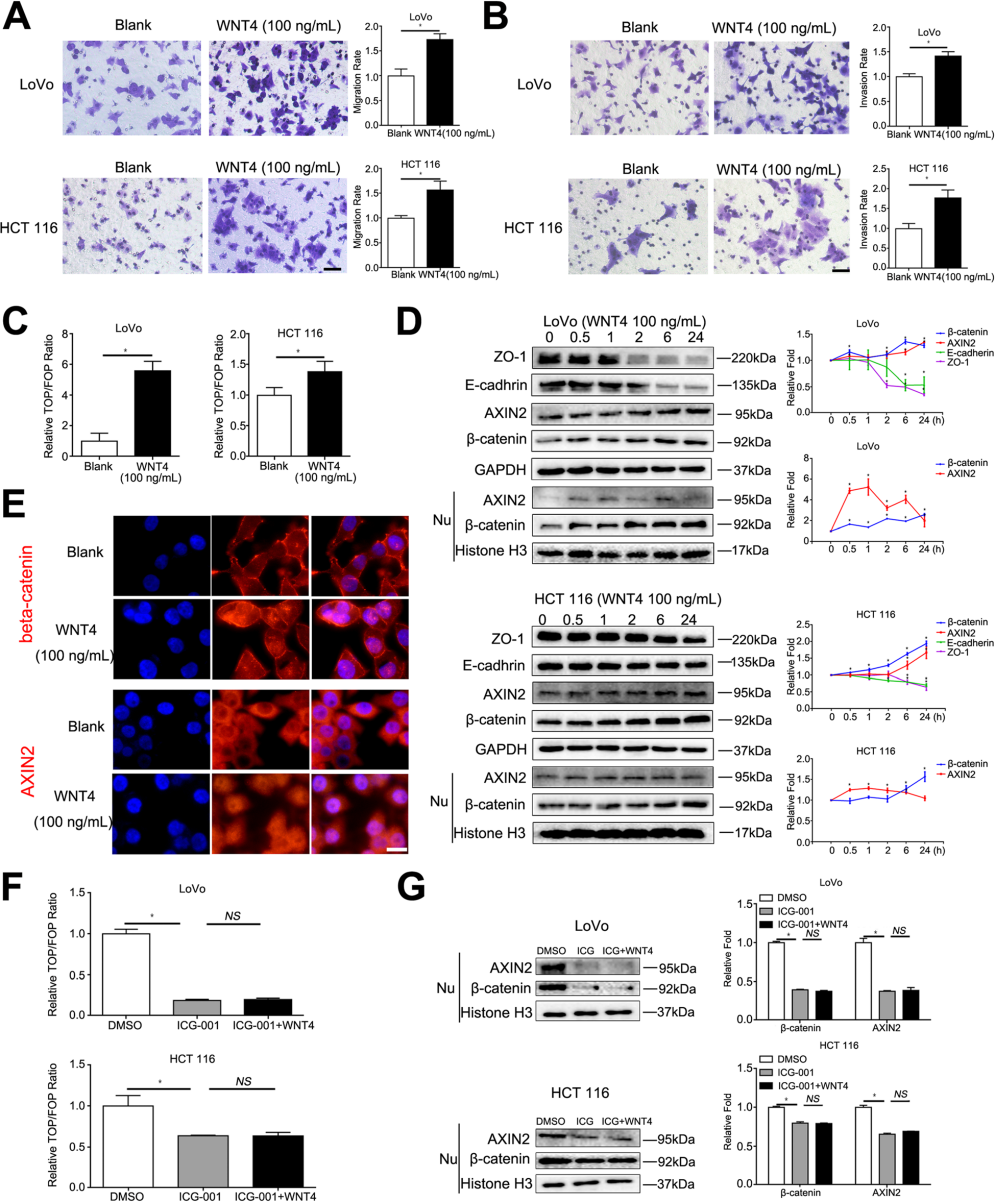
WNT4 induces the expression of β-catenin. **a** and **b** Cell migration and invasion capabilities of colorectal cancer (CRC) cells pretreated with WNT4 (100 ng/mL) for 24 h. Scale bar, 50 μm. **c** TOP-flash plasmid or FOP-flash (2 mg) transfected into the CRC cells for 48 h. Cells were treated with WNT4 (100 ng/mL) for 6 h before analysis. **d** LoVo and HCT 116 CRC cells treated with WNT4 (100 ng/mL); and the expression of β-catenin, AXIN2, E-cadherin, ZO-1, and nuclear β-catenin and AXIN2 were determined by western blot analysis. **e** Higher expression and nuclear translocalization of β-catenin and AXIN2 were shown in immunofluorescence after LoVo cells treated with WNT4 (100 ng/mL) for 6 h. Scale bar, 10 μm. **f** and **g** TOP/FOP-Flash reporter assay and western blots were used to detect whether the WNT pathway was activated. CRC cells were treated with ICG-001 (25 μM) for 24 h and WNT4 (100 ng/mL) for 6 h. Two-tailed Student’s t test and ANOVA were used for statistical analyses. All experiments were performed in triplicate. Measurement data were presented as the mean ± SD. *P < 0.05; NS, no significance

Since we found that WNT4 was related to the clinical characteristics of CRC, we further explored the effect of WNT4 on CRC. Migration and invasion effects of WNT4 on CRC were measured after adding exogenous WNT4. Exogenous WNT4 at a concentration of 100 ng/mL significantly enhanced the migration and invasion ability of LoVo and HCT 116 cells (Fig. 2a, b, *P* < 0.05).

To further analyze the mechanism of WNT4 that promoted invasion and migration, TOP/FOP Flash assays were applied to evaluate effect of WNT4 on the Wnt/β-catenin signaling pathway in CRC cells. TOP/FOP Flash assays are a direct proof to test β-catenin-mediated transcriptional activity. E-cadherin and ZO-1 are two markers known to be involved in EMT were also measured [19, 20]. The TCF/LEF transcription activity was significantly enhanced after treatment with WNT4 (100 ng/mL) for 6 h (Fig. 2c, *P* < 0.05), thereby indicating that the Wnt/β-catenin signaling pathway was activated. Next, we found that total levels of β–catenin and AXIN2, a downstream target gene of β–catenin, were increased, while the levels of E-cadherin and ZO-1 were decreased in LoVo and HCT 116 cells treated with exogenous WNT4 (100 ng/mL) for 0, 0.5, 1, 2, 6, and 24 h, (Fig. 2d, *P* < 0.05). Moreover, the level of β-catenin in the nucleus also increased (Fig. 2d, *P* < 0.05), while levels of AXIN2 were the highest at 1 h after treatment. Fluorescence microscopy also demonstrated that CRC cells treated with WNT4 increased β–catenin and AXIN2 accumulation and remarkably induced nuclear translocation of β–catenin and AXIN2 (Fig. 2e). To further verify that WNT4 activated downstream pathways through the canonical Wnt/β–catenin pathways, an inhibitor of transcription mediated by β-catenin/TCF, ICG-001 was used. As shown in Fig. 2f and g, 25 μM ICG-001 completely inhibited the activation of β-catenin signaling caused by WNT4 (*P* < 0.05).

**Fig. 3:**
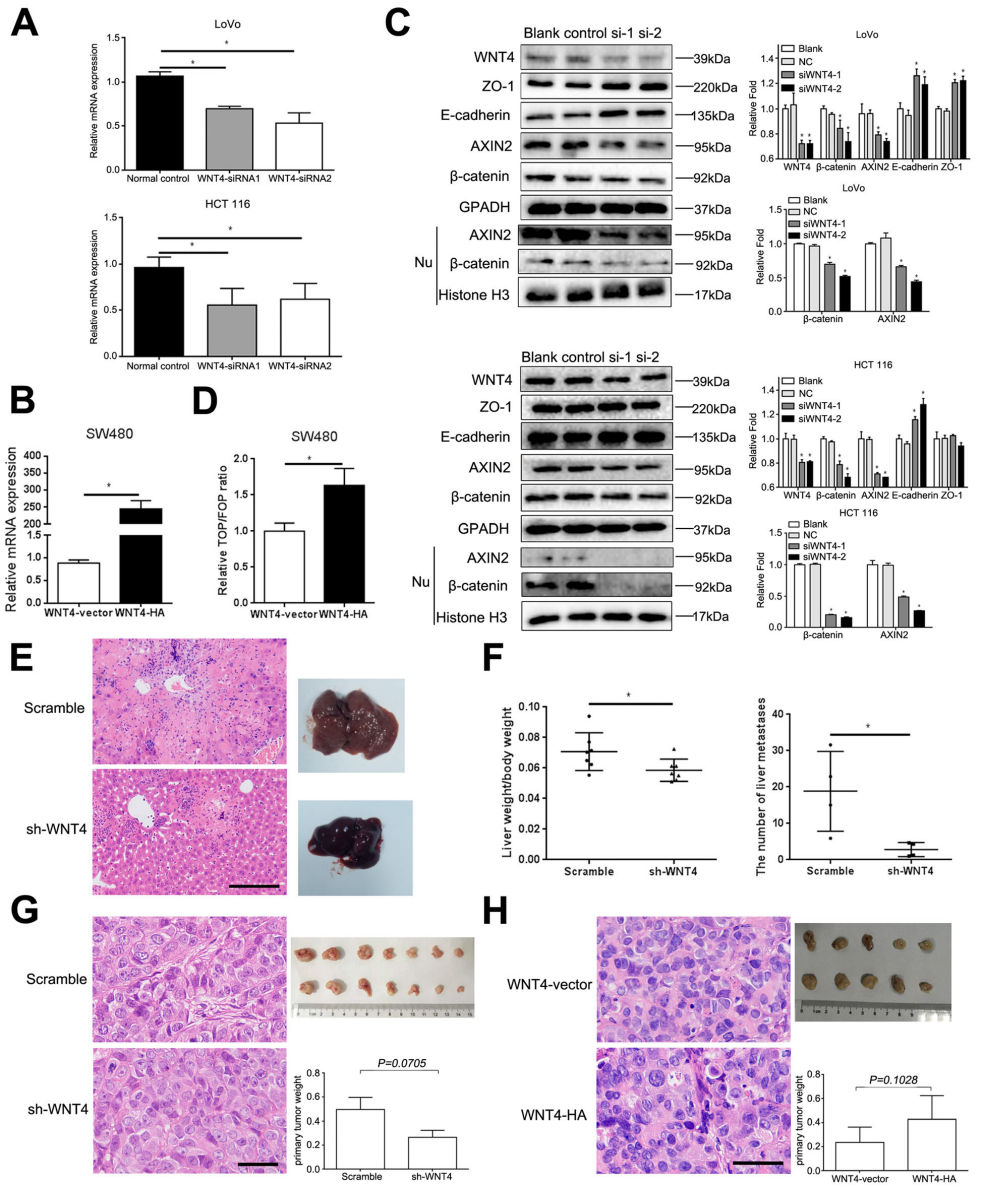
WNT4 can promotes metastasis of colorectal cancer cells. **a** The mRNA level of WNT4 in colorectal cancer (CRC) cells transfected with WNT4-siRNA1, WNT4-siRNA2, and normal control. **b** The mRNA level of WNT4 in SW480 cells transfected with WNT4-vector and WNT4-HA. **c** CRC cells were transfected with WNT4-siRNA1, WNT4-siRNA2, then, WNT4, β-catenin, AXIN2, E-cadherin, ZO-1, and nuclear β-catenin and AXIN2 were analyzed by western blot. **d** Overexpression of WNT4 enhanced the TCF/LEF transcription activity. **e** and **f** Tumor cell metastasis assay was performed in vivo; the ratio of liver weight to body weight and the number of liver metastases in the Scramble group are much higher than the WNT4-shRNA group (*n* = 7). Scale bar, 50 μm. **g** and **h** A subcutaneous xenograft model in nude mice was used to identify if WNT4 promoted the proliferation of colon cancer cells (LoVo cells, *n* = 7; SW480 cells, *n* = 5). No significant difference was observed. **g** Scale bar, 20 μm. **h** Scale bar, 50 μm. Data are presented as the mean ± SD. Two-tailed Student’s t test was used for statistical analyses. *P < 0.05

In addition, CRC cells expressing different levels of WNT4 were also constructed. LoVo and HCT 116 cells highly expressed WNT4 while only low levels of WNT4 were found in SW480. Therefore, LoVo, HCT 116, and SW480 cells were chosen for the following experiments. HCT 116 and LoVo cells transfected with WNT4-siRNA1, WNT4-siRNA2 and normal control siRNA (normal control) were constructed, respectively (Fig. 3a, *P* < 0.05). After validating the knockdown effects of WNT4-siRNAs in LoVo and HCT 116 cells, we successfully constructed a stably transfected WNT4-vector and WNT4-HA in SW480 cells (Fig. 3b, *P* < 0.05). We found that the total levels of β–catenin and AXIN2 in these cells were significantly decreased, while the levels of E-cadherin and ZO-1 were increased in both loVo and HCT 116 cells transfected WNT4-siRNA1 and WNT4-siRNA2 (Fig. 3c, *P* < 0.05). Meanwhile, the levels of β-catenin and AXIN2 in the nucleus also decreased (Fig. 3c, *P* < 0.05). Next, the TCF/LEF transcription activity in SW480 cells overexpressed WNT4 (WNT4-HA) significantly enhanced (Fig. 3d, *P* < 0.05). Taken together, these results may suggest that WNT4 promoted the migration and invasion of CRC by promoting EMT through the β-catenin-dependent pathway.

### WNT4 promotes invasion and migration of CRC through β-catenin-dependent pathway in vivo

To confirm our results in vitro, we then conducted a series of in vivo experiments. LoVo cells (1× 10^6^) stably transfected with negative control shRNA (Scramble) or WNT4-shRNA were injected into the caudal vein of nude mice (*n* = 7), respectively. Since liver metastasis is the most common distant metastasis of CRC, we determined the number of metastasis sites in the liver of those mice. Our data showed that liver metastasis occurred in four mice in each group. The ratio of liver weight to body weight and the number of liver metastases in the Scramble group were much higher when compared to the WNT4-shRNA group (Fig. 3e, f, *P* < 0.05). These results suggested that WNT4 may be a key player in promoting CRC metastasis.

To further explore if WNT4 had a pro-proliferation effect on CRC in vivo, a subcutaneous xenograft model was established in nude mice. The results showed that tumors developed from mice incubated with LoVo cells (1 × 10^7^ cells/200 μL) transfected with WNT4-shRNA were smaller and lighter than those incubated with LoVo cells transfected with negative control shRNA (Scramble), however, the difference was not statistically significant (Fig. 3g, *P* = 0.0705, *n* = 7). In mice incubated with SW480 cells (1 × 10^7^ cells/200 μL) stably transfected WNT4-vector and WNT4-HA, tumors developed from WNT4-HA were larger and heavier compared to those developed from WNT4-vector cells, while no significant difference was observed (Fig. 3h, *P* = 0.1028, *n* = 5). Taken together, these results may indicate that WNT4 only has a slight effect on tumor proliferation.

### WNT4 promotes fibroblast recruitment and activation via β-catenin-dependent pathway

**Fig. 4:**
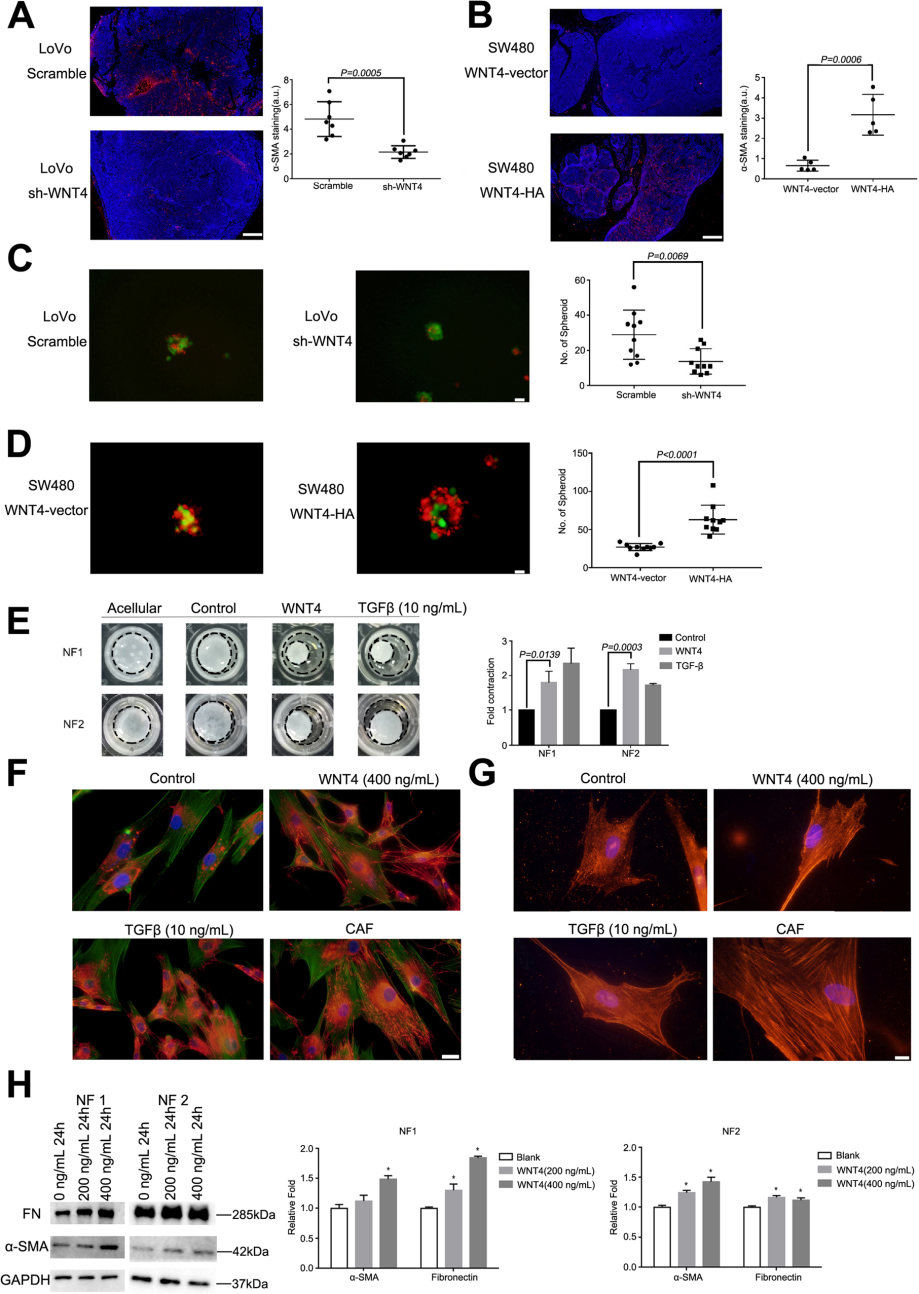
WNT4 promotes the recruitment and activation of fibroblasts. **a** and **b** The expression of infiltrating α-SMA-positive cancer associated fibroblasts (CAFs) in different groups: LoVo-Scramble or LoVo-shWNT4 (*n* = 7), SW480-WNT4-vector or SW480-WNT4-HA (*n* = 5). Scale bar, 200 μm. CAFs, red; DAPI, bule. **c** and **d** Typical heterospheroids formed in different groups: LoVo-Scramble or LoVo-shWNT4, SW480-WNT4- vector or SW480-WNT4- HA. CAF, red; CRC cells, green; Scale bar, 20 μm. **e** Stronger gel contraction was observed after the NF#1 and NF#2 treatment with WNT4 (400 ng/mL). **f** and **g** Increased expression of fibronectin (FN) and α-SMA were observed by immunofluorescence. CAFs and NFs treated with TGF-β (10 ng/mL) served as positive controls. **f** fibronectin-red, phalloidin-green, scale bar, 50 μm. **g** α-SMA-red, scale bar, 20 μm. **h** Increased expression of FN and α-SMA were observed by western blotting analysis. A two-tailed Student’s t test was used for statistical analyses. Measurement data were presented as the mean ± SD. *P < 0.05

Despite secreted WNT4 initiating EMT, as illustrated above, the effect of highly secreted WNT4 levels in the CRC microenvironment remains largely unknown. Activated Wnt/β-catenin signaling has been implicated in fibrosis in a number of organs, including tumor stroma [21–23]. However, it is not clear whether WNT4 can also activate fibroblasts through β-catenin signaling. Strikingly, we found that α-SMA positive CAFs were more abundant in tumors from mice injected with the LoVo-Scramble (*n* = 7) and SW480-WNT4-HA-transfected cells (*n* = 5) when compared to those from mice injected with the LoVo-shWNT4 and SW480-WNT4-vectors, respectively (Fig. 4a, b, *P* = 0.0005, *P* = 0.0006, respectively). These results indicated that WNT4 may contribute to CRC tumor progression by recruiting and activating fibroblasts.

To investigate if WNT4 could recruit fibroblasts, NFs and CAFs were isolated and identified from the tumor and adjacent tissues of CRC patients by an enzymatic dissociation method. Cell surface markers CD31, CD45, and CD329 were used to confirm the absence of endothelial, immune, and epithelial cell contamination by flow cytometry analysis (Supplementary Fig. 1). We established a suspension coculture of CAFs and tumor cells with complete medium in ultra-low attachment plates. Intriguingly, fewer typical heterospheroids were formed in the WNT4-shRNA groups, thereby supporting the hypothesis that WNT4 could recruit fibroblasts and promote the adhesive capacity of fibroblasts to CRC cells (Fig. 4c, *P* = 0.0069). Moreover, in the WNT4-HA group, more heterospheroids were found compared to the WNT4-vector group, which further confirmed our hypothesis (Fig. 4d, *P* < 0.0001).

In addition, exogenous WNT4 was added to the growth medium (without FBS) of NFs at a final concentration of 0 (control) or 400 ng/mL for 24 h. Stronger gel contraction was observed after the NFs treated with WNT4, indicating increased extracellular matrix remodeling (Fig. 4e, *P* = 0.0139, *P* = 0.0003, respectively). We also tested markers of CAFs, fibronectin (FN) and α-SMA by immunofluorescence assay and western blotting analysis to verify the recruitment and contraction ability of WNT4 towards fibroblasts. Increased expression of FN and α-SMA were observed after NFs were stimulated with WNT4 (400 ng, 24 h) (Fig. 4f, g, h, *P* < 0.05). Together, these data highlight WNT4 as a novel and highly potent factor that drives conversion of NFs into CAFs within the tumor microenvironment. We next investigated the underlying mechanism of how WNT4 could activate NFs into CAFs.

**Fig. 5:**
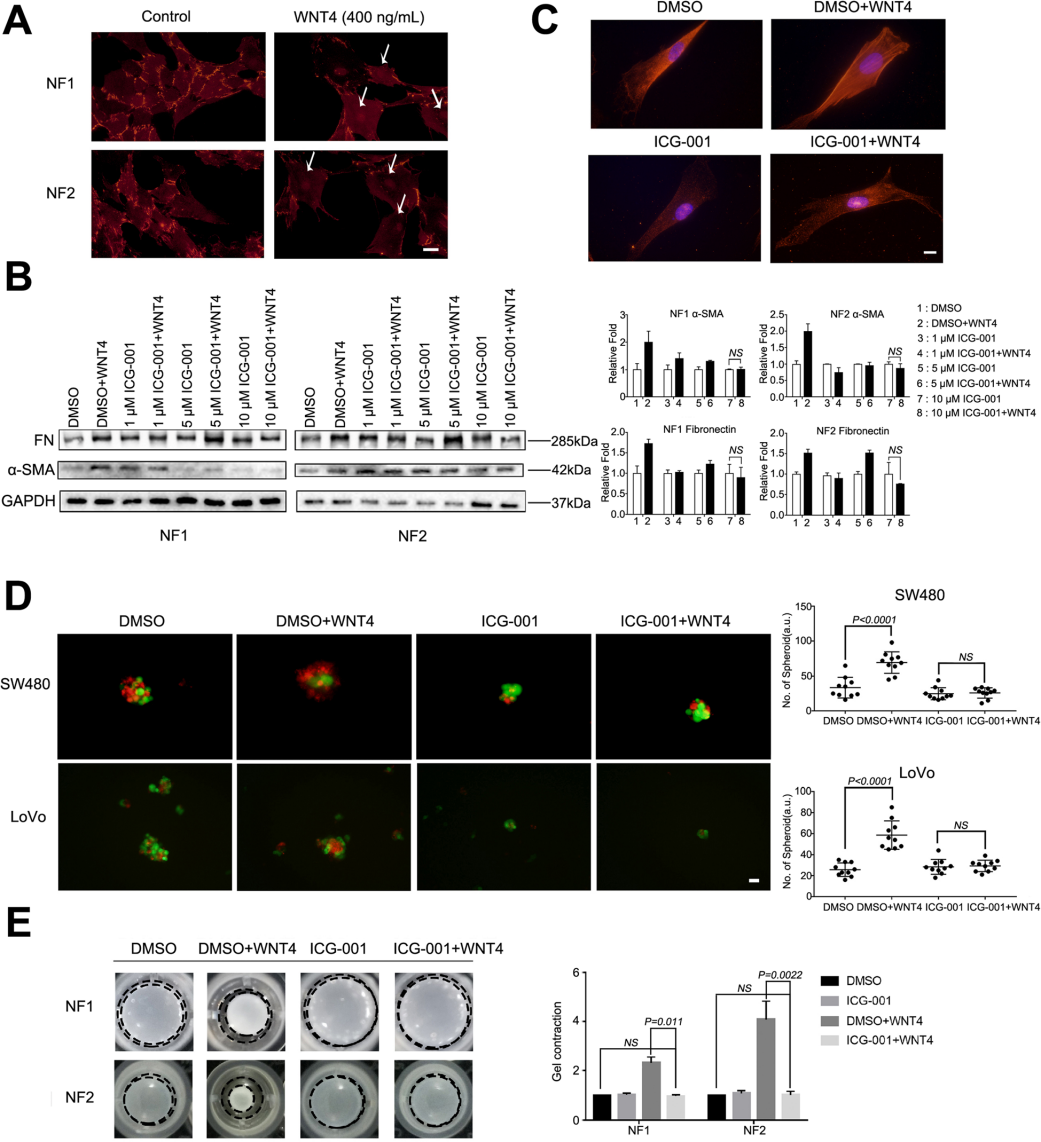
WNT4 signaling in fibroblasts. **a** β-catenin translocation from the cytoplasm to the nucleus in the NF#1, NF#2 treated with WNT4 protein (400 ng/ml, 24 h). Scale bar, 50 μm. **b** and **c** ICG-001 completely blocked elevation of α-SMA and fibronectin at 10 μM was detected by western blot. Scale bar, 20 μm. **d** and **e** The effect of WNT4 on the formation of heterospheroids and gel contraction ability was completely inhibited by ICG-001 (10 μM). Scale bar, 20 μm. Measurement data were presented as the mean ± SD. *P < 0.05; NS, no significance

The WNT/β-catenin pathway is known to play an important role in CRC-associated fibroblasts [24, 25]. To interrogate the WNT4 signaling pathways operating in CAF activation, we first assessed activation of the β-catenin-dependent pathway using immunofluorescence assay. As shown in Fig. 5a, β-catenin nuclear translocation was observed in NFs treated with WNT4 (400 ng/ml, 24 h). These data suggested that WNT4 could activate the classical WNT/β-catenin pathway in colorectal NFs. Subsequently, ICG-001 was used to block the canonical β-catenin pathway. We found that ICG-001 completely blocked the expression of α-SMA and FN at a concentration of 10 μM (Fig. 5b, c, *P* < 0.05). Additionally, the effects of WNT4 protein on heterospheroid formation and gel contraction ability were also completely inhibited by ICG-001 (Fig. 5d and e, *P* < 0.05).

### WNT4 promotes angiogenesis in CRC via the WNT4/β-catenin/ANG2 pathway

**Fig. 6:**
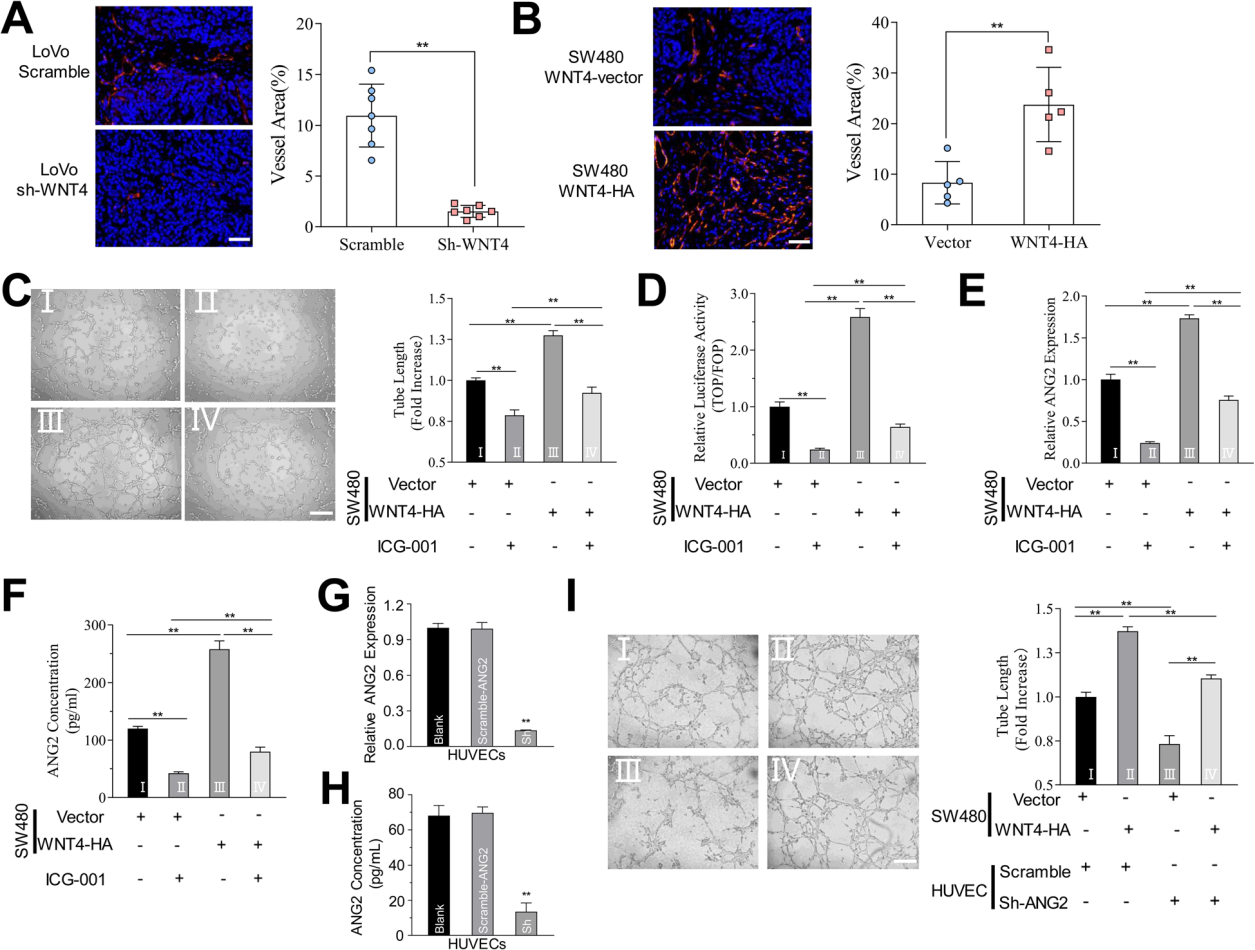
WNT4 promote angiogenesis in colorectal cancer. **a** and **b** The expression of CD31 (red) in different groups: LoVo-Scramble or LoVo-shWNT4 (**a**), SW480-WNT4-vector or SW480-WNT4-HA. Scale bar, 50 μm. **c** The ability of tube formation was significantly improved when HUVECs were treated with conditioned media (CM) from SW480 cells transfected with WNT4-HA than that from SW480 transfected WNT4-vector, and it could be blocked by the β-catenin/TCF inhibitor ICG-001 (10 μM). Scale bar, 100 μm. **d** TOP/FOP-Flash reporter was used to detect whether the WNT pathway was activated. **e** and **f** The levels of ANG2 were detected by RT-qPCR and ELISA in HUVECs that underwent different treatments. **g** and **h** The mRNA level of ANG2 in cells and CM of HUVECs transfected with negative control shRNA (Scramble) and sh-ANG2 were detected by RT-qPCR. **i** The ability of tube formation promoted by high WNT4 expression was significantly inhibited due to low ANG2 expression. Scale bar, 100 μm. **P < 0.01

Angiogenesis is critical in tumor development, therefore, we next studied whether WNT4 could promote angiogenesis in CRC. In subcutaneous xenograft mouse model, higher levels of endothelial (CD31) were found in tumors from mice injected with LoVo cells transfected Scramble and SW480 cells transfected WNT4-HA compared to that from mice injected with LoVo cells transfected WNT4-shRNA and SW480 cells transfected WNT4-vector, respectively (Fig. 6a, b, *P* < 0.05). Thus, these results indicated that WNT4 may promote angiogenesis in CRC. The underlying mechanism involved remains to be elucidated.

To investigate the possible role of WNT4 in angiogenesis, HUVECs were treated with CM from CRC cells with different levels of WNT4, and the effects of WNT4 on the ability in generating tubular networks of HUVECs on Matrigel were evaluated. Our results showed that tube formation was significantly increased when HUVECs were treated with CM from SW480 cells transfected with WNT4-HA when compared to that from SW480 transfected WNT4-vector (Fig. 6c, *P* < 0.05). These results indicated that WNT4 could promote angiogenesis.

To explore whether WNT4 promoted tumor angiogenesis via the β-catenin pathway, the TOP/FOP Flash assay was used to determine TCF/LEF transcription activity when HUVECs were treated with CM from SW480 cells with different expression levels of WNT4 (as described above). HUVECs treated with CRC cells with high WNT4 expression resulted in upregulation of transcriptional activity (Fig. 6d, *P* < 0.05). Subsequently, we found that β-catenin transcriptional activity was downregulated, and tube formation was significantly abrogated after blocking with ICG-001 (10 μM) (Fig. 6c, d, *P* < 0.05). These results demonstrated that WNT4 could promote angiogenesis via the β-catenin-dependent pathway.

We next measured the expression of ANG2 to investigate whether ANG2 could be regulated by the WNT4/β-catenin pathway. RT-qPCR and ELISA were used to determine the expression of ANG2 from different treated HUVECs (Fig. 6e, f, *P* < 0.05). We can infer that ANG2 was upregulated with high levels of WNT4, and was downregulated after blocking with ICG-001 (10 μM) (Fig. 6e, f, *P* < 0.05). In addition, HUVECs stably transfected with negative control shRNA (Scramble) and sh-ANG2 were constructed, and the expression of ANG2 was determined by RT-qPCR and ELISA (Fig. 6g, h, *P* < 0.01). Tube formation promoted by high WNT4 expression was significantly inhibited due to low ANG2 expression (Fig. 6i, *P* < 0.01). These findings indicated that angiogenesis could be regulated by the WNT4/β-catenin/ANG2 cascade.

### WNT4 levels can be regulated by miR-497

**Fig. 7:**
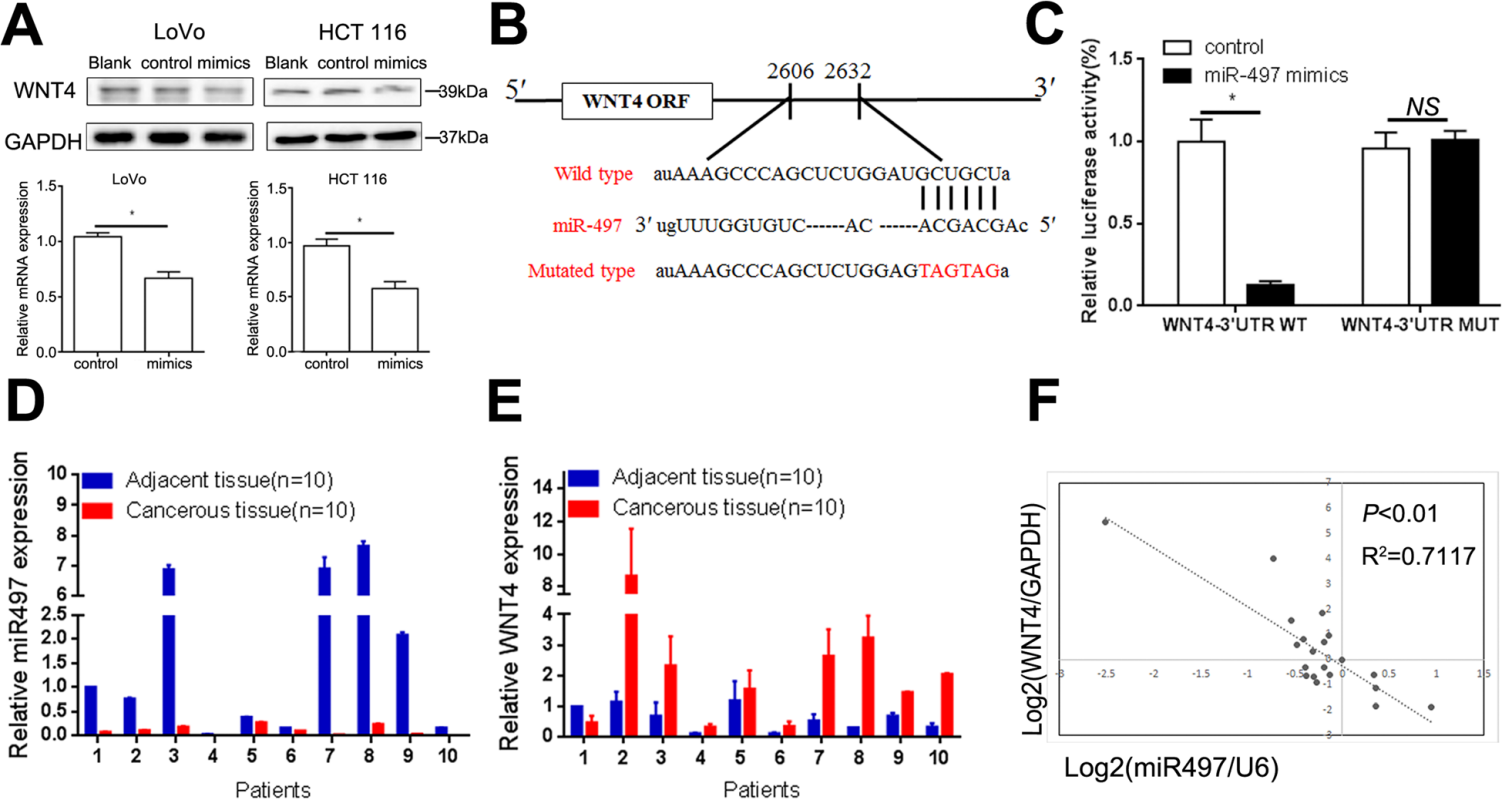
WNT4 levels can be regulated by miR-497. **a** Western blotting assays and RT-qPCR showed the protein and mRNA expression of WNT4 in CRC cells after transfection with miR-497 mimics. **b** The target sites of miR-497 in the 3′-UTR of WNT4 shown as a schematic representation. **c** Wild-type or mutant 3′-UTR constructs of WNT4 were cloned into pMIR-REPORT™ vectors, respectively, and co-transfected with miR-497 mimics in LoVo cells. **d** and **e** The mRNA level of miR-497 and WNT4 in the paired colorectal cancer tissues and adjacent tissues. **f** An inverse correlation was found between miR-497 expression and WNT4 in 10 paired CRC tissues and adjacent normal tissues (Spearman’s correlation, *P* < 0.01, *R*^2^ = 0.7117)

MiR-497 has been well characterized as a tumor suppressor in our previous study [26]. We found that WNT4 expression was decreased at both mRNA and proteins levels in LoVo and HCT 116 CRC cells after transfecting with miR-497 mimics (Fig. 7a, *P* < 0.05). To further determine the relationship between miR-497 and WNT4, a Dual-Luciferase Reporter Assay was conducted (Fig. 7b). We found that miR-497 suppressed the reporter gene activity of WNT4 in LoVo cells (Fig. 7c, *P* < 0.05), whereas mutant plasmids did not show any change in reporter gene activity (Fig. 7c, *P* > 0.05). Next, the expression of miR-497 and WNT4 among 10 randomly selected CRC tissues and paired adjacent tissues were investigated by quantitative real-time PCR (qRT-PCR). Significant downregulation of miR-497 and upregulation of WNT4 expression were found (Fig. 7d, e). Moreover, a negative correlation was observed between miR-497 and the WNT4 expression levels in CRC cancer tissues and paired adjacent tissues (Fig. 7f, *P* < 0.01). Taken together, these data suggested that miR-497 could downregulate WNT4 levels.

## Discussion

WNT ligands have frequently been reported to play an important role in CRC, but the role of WNT4 on CRC has rarely been studied. In a previous study, it has been reported that WNT4 promoted the proliferation of breast cancer stem cells [27], and promoted progression of gastric cancer [28], which suggested a pro-carcinogenic role of WNT4. However, these studies focused on studying the expression of Wnt ligands in tissues samples [29, 30], while the serum levels of WNT4 has not been identified previously. In the present study, we demonstrated for the first time that WNT4 was elevated in the serum of CRC patient and originated from tumor tissues by measuring the CM from colorectal tissue culture, and was decreased after tumor resection. Besides, analysis of the relationships between serum levels of WNT4 and clinicopathological characteristics showed that elevated WNT4 correlated with the advanced stage and metastasis of CRC. These findings indicated that WNT4 may be a potential biomarker for CRC. The detection of tumor-associated biomarkers in peripheral blood of cancer patients provides an opportunity to analyze the changes in tumor burden and monitor the response to treatment [31]. The results obtained in this study suggested that WNT4 could potentially be a serum marker for diagnosis, and value the risk of metastasis for CRC.

Moreover, when compared to CEA and CA199 levels in serum, we found that serum levels of WNT4 had significantly higher sensitivity and specificity for detection of CRC. However, due to the lack of a robustly designed prospective trial, these results remain subject to further verification. Taken together, our results highlight the potential clinical significance of WNT4, and suggest that further studies are needed to investigate the diagnostic applications of WNT4 in CRC.

Although the upregulation of WNT expression has been extensively investigated in CRC and corresponding liver metastasis [32–34], the role of WNT4 in CRC has rarely been explored. To investigate the effect of elevated WNT4 levels in CRC tissues, exogenous and endogenous WNT4 was used to treat CRC cells, fibroblasts and HUVECs. In our study, we demonstrated that WNT4 could promote EMT of CRC cells through the WNT4/β-catenin cascade. Cancer cells often required mesenchymal phenotype to enhance their ability of invasion and metastasis [35], which is consistent with our finding that WNT4 could promote EMT in CRC cells to promote the invasion and migration ability both in vitro and in vivo. The canonical Wnt signalling pathway regulates target genes via stabilization of nuclear β-catenin [36]. As evidently supported that Axin2 is a part of destruction complex and could captures and phosphorylates β-catenin in the absence of Wnt [16, 37]. In our study, we found the interesting phenomenon that total levels of β-catenin and Axin2 protein were reduced following WNT4 knockdown in both cell lines, which may infer that WNT4 may could also regulate β-catenin through a non-canonical pathway, and Axin2 may be regulated by WNT4. Previous study also demonstrated that the putative tumor suppressor Axin2 is also upregulated in CRC, and they proved that Axin2 could upregulate the activity of Snail1(a transcriptional repressor), thereby inducing a EMT and driving metastatic activity in CRC [38]. Thus, it would be interesting to elucidate the potential mechanism involved.

Furthermore, most studies have focused on the role of WNT ligands in cancer cells, the Wnt/β-Catenin pathway, for example, could promote gastric cancer cells [39], non-small cell lung cancer [40] and CRC cells [41]. However, little attention has been paid to its possible role in the tumor microenvironment, including CAFs and endothelial cells. In a previous study, it was demonstrated that WNT2 protein could be secreted by fibroblasts to promote the progression of esophageal cancer [42]. In this study, we firstly showed that WNT4 secreted by CRC cells could activate surrounding fibroblasts through the WNT4/β-catenin pathway. Furthermore, recent evidence suggests a significant role for EMT in fibrosis, including its involvement in tumor stroma activation [24, 43]. In the current study, we showed that WNT4 prompted the nuclear translocation of β-catenin and increased the expression of α-SMA and fibronectin in NFs, which subsequently enhanced reinforced cell contraction and microsphere formation. We next found that WNT4 secreted by CRC cells could enhance angiogenesis in CRC. ANG2 iss a critical gene for angiogenesis, however, the correlation of ANG2 and WNT4/β-catenin has never been investigated before. In our study, we demonstrated that angiogenesis could be activated by WNT4 through the WNT4/β-catenin/ANG2 pathway, which is vital for tumor invasion and metastasis. To our knowledge, few studies have explored the effect of WNT4 on those components in the CRC microenvironment. In a previous study, it was shown that WNT4 could be released into the stroma in CRC in the form of exosomes [44]. It has been widely reported that exosomes from CRC cells play critical role in modulating invasion, immune responses and angiogenesis [45]. Therefore, we speculated that WNT4 could be secreted as exosomes from CRC cells and act on various components in the tumor microenvironment to promote tumor progression. Additional studies will be required to support this hypothesis.

It is worth noting that these effects of WNT4 were observed at a concentration that has been reported in previous studies [46, 47], while no effect was observed at serum concentration. Combined with our results, we speculated that WNT4 secreted by CRC tissue mainly acted on the tumor microenvironment to create an environment that is conducive to tumor growth, while WNT4 in serum may simply act as a diagnostic or prognostic indicator for CRC.

In addition, we found that downregulation miR-497 may lead to elevated WNT4 in CRC tissues. Downregulation of miR-497 and its tumor-suppressive role have been reported in multiple cancers [48–51]. Our data showed that WNT4 was a novel direct target of miR-497. However, further studies are required to confirm the regulatory effect of miR-497 on WNT4 expression and its role in the progression of CRC.

## Conclusion

In our study, we demonstrated for the first time that WNT4 could be a potential biomarker for CRC, which could be used to diagnose and value the risk of metastasis for CRC. Our data then substantiated the effects of secretory WNT4 on the development of the colorectal tumor microenvironment. WNT4 promoted CRC progression and liver metastasis through the activation of WNT4/β-catenin signaling. EMT could be induced by WNT4 to promote the migration of CRC cells via WNT4/β-catenin signaling. Moreover, fibroblasts were activated by WNT4 through WNT4/β-catenin signaling and transformed from NFs into CAFs. This increased the number of CAFs in the tumor stroma and further promoted the metastasis of CRC cells. Moreover, angiogenesis induced by WNT4/β-catenin/ANG2 signaling also promoted metastasis of CRC. In conclusion, in the present study, we revealed that WNT4 is a novel molecule involved in CRC progression, which may help aid in the diagnosis and treatment of CRC.

## Supplementary Information

**Additional file 1:** **Supplementary Table 1.** The sequences of siRNAs and shRNA used in the study.

**Additional file 2:** **Supplementary Table 2.** The primers used in the study.

**Additional file 3:** **Supplemental Figure 1.** To identify fibroblasts, specific cell surface markers CD31, CD45 and CD329 were used to confirm the absence of endothelial, immune, and epithelial cell contamination by flow cytometry analysis.

## Data Availability

All remaining data are available within the article and supplementary files, or available from the authors upon request.

## Abbreviations

BSA: Bovine serum albumin
CRC: Colorectal cancer
CAF: Cancer associated fibroblast
EMT: Epithelial-to-mesenchymal transition
IHC: Immunohistochemical staining
NF: Normal fibroblast
shRNA: Short hairpin RNA
siRNA: Small Interference RNA
RT-qPCR: Real-time quantitative polymerase chain reaction
