# Supplementary material for: WNT4 secreted by tumor tissues promotes tumor progression in colorectal cancer by activation of the Wnt/β-catenin signalling pathway
Source: J Exp Clin Cancer Res. 2020 Nov 23;39:251. doi: 10.1186/s13046-020-01774-w (PMC7682076; doi:10.1186/s13046-020-01774-w)
Supplement: Supplementary file 1 — Additional file 1: Supplementary Table 1. The sequences of siRNAs and shRNA used in the study. [file 13046_2020_1774_MOESM1_ESM.docx]

**Supplementary Table 1. The sequences of siRNAs and shRNA used in the study**

| si-RNA Sence(5′-3′) antisence(5′-3′) |
| --- |
| si-WNT4-1 UCCACACUCGACUCCUUGCTT GCAAGGAGUCGAGUGUGGAGC  si-WNT4-2 AGGAGACGUGCGAGAAACUTT AGUUUCUCGCACGUCUCCUCC  sh-ANG2 GCAACGCUAUGUGCUUAAATT UUUAAGCACAUAGCGUUGCTT  miR-497 mimics CAGCAGCACACUGUGGUUUGU AAACCACAGUGUGCUGCUGUU  control UUCUCCGAACGUGUCACGUTT ACGUGACACGUUCGGAGAATT |
