# Supplementary material for: WNT4 secreted by tumor tissues promotes tumor progression in colorectal cancer by activation of the Wnt/β-catenin signalling pathway
Source: J Exp Clin Cancer Res. 2020 Nov 23;39:251. doi: 10.1186/s13046-020-01774-w (PMC7682076; doi:10.1186/s13046-020-01774-w)
Supplement: Supplementary file 2 — Additional file 2: Supplementary Table 2. The primers used in the study. [file 13046_2020_1774_MOESM2_ESM.docx]

**Supplementary Table 2. The primers used in the study.**

| Gene name Primer sequence (5′-3′) |
| --- |
| U6 snRNA F: GGAACGATACAGAGAAGATTAGC  R: TGGAACGCTTCACGAATTTGCG  miR-497 F: CAGCAGCACACTGTGGTTTGTA  R: Uni-miR qPCR primer  GAPDH F: GGAAGCTTGTCATCAATGGAAATC  R: TGATGACCCTTTTGGCTCCC  WNT4 F: TCACGCACTGAAGGAGAAGT  R: TGGACGTCTTGTTGCATGTG  ANG2 F: CAGAGGCTGCAAGTGCTGGAGAACA  R: GAGGGAGTGTTCCAAGAGCTGAAGT |
