# Supplementary figures and images for: WNT4 secreted by tumor tissues promotes tumor progression in colorectal cancer by activation of the Wnt/β-catenin signalling pathway
Source: J Exp Clin Cancer Res. 2020 Nov 23;39:251. doi: 10.1186/s13046-020-01774-w (PMC7682076; doi:10.1186/s13046-020-01774-w)

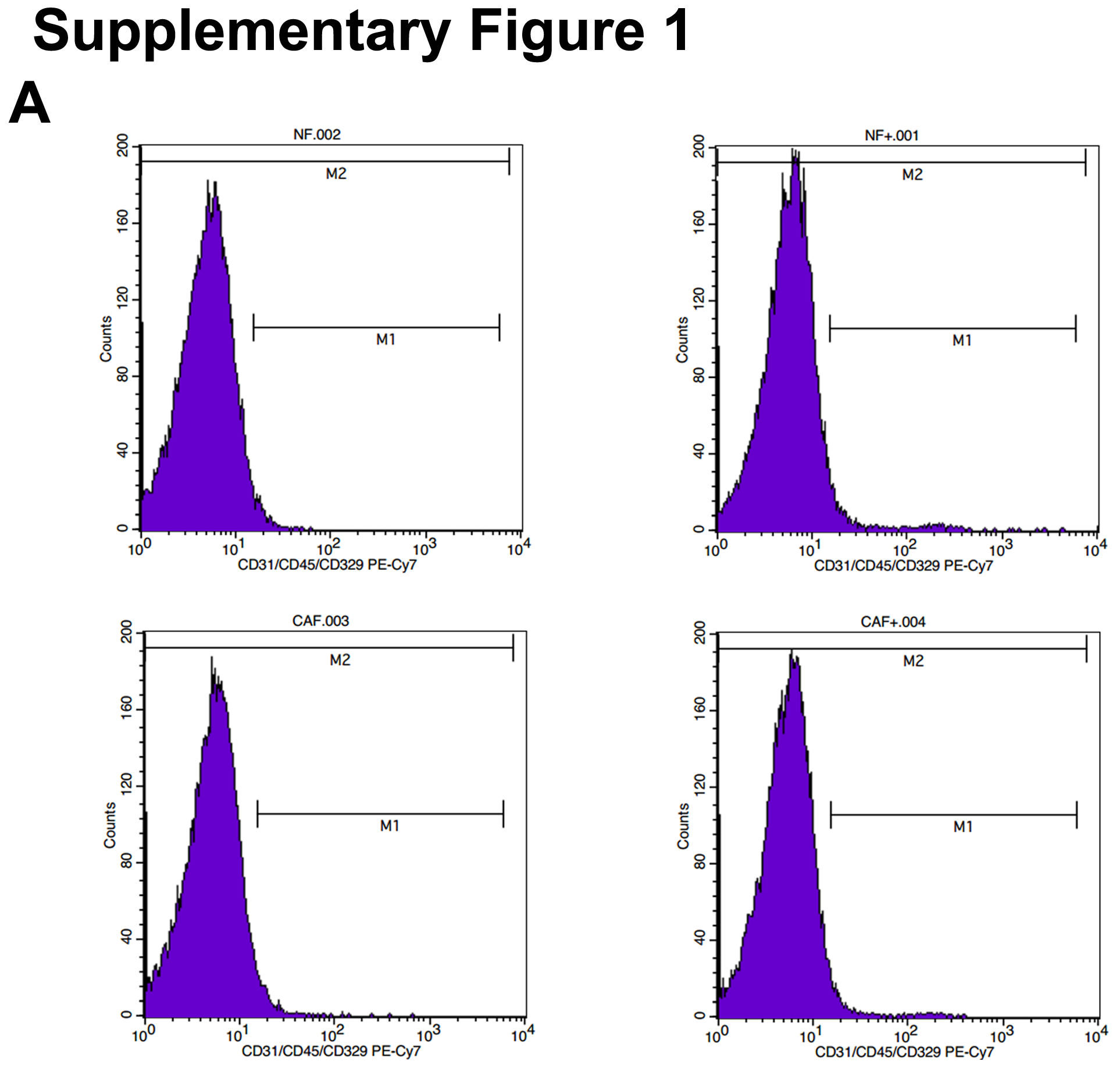

Supplement: Supplementary file 3 — Additional file 3: Supplemental Figure 1. To identify fibroblasts, specific cell surface markers CD31, CD45 and CD329 were used to confirm the absence of endothelial, immune, and epithelial cell contamination by flow cytometry analysis. [file 13046_2020_1774_MOESM3_ESM.png]
